# Self-Concept in Children with Primary Nocturnal Enuresis and Related Influencing Factors

**Published:** 2020-04

**Authors:** Yanli MA, Ying SHEN, Xiaomei LIU

**Affiliations:** 1. Department of Nephrology, Beijing Children’s Hospital, Capital Medical University, Beijing, China; 2. Beijing Children’s Key Laboratory of Chronic Kidney Disease and Blood Purification, Beijing, China; 3. General Ward, Beijing Children’s Hospital, Capital Medical University, Beijing, China

## Dear Editor-in-Chief

Enuresis is a very common developmental disorder which refers to intermittent incontinence occurring in children aged 5 yr old and above during periods of sleep (1). Primary Nocturnal Enuresis (PNE) occurs in children who have never been consistently dry throughout the night (2). Although it does not cause any physical limitations in the life of children, enuresis is one of the most common chronic problems which could cause psychological disorders, such as behavioural problems, attention-deficit hyperactivity disorders (3), and anxiety disorders. To our knowledge, the relationship between self-perception and enuresis remains controversial and needs further investigation.

The aim of this study was to compare children diagnosed with PNE with their normal peers in terms of self-concept. Moreover, the possible related factors of decreased self-concept were analyzed.

This study was approved by the Institutional Review Board of Beijing Children’s Hospital. All parents signed informed consent.

Children aged 8 to 15 yr old diagnosed with PNE from October 2016 to April 2018 in Beijing Children’s Hospital, Beijing, China, were included in this study. The control group was composed of age- and gender-matched children who attended primary schools and middle schools in the city of Beijing. The Piers-Harris Children’s Self-Concept Scale (PHCSS) with good validity and reliability includes six dimensions: behavior, intellectual and school status, physical appearance and attributes, anxiety, popularity, and happiness and satisfaction (4). The questionnaires were distributed within the same day in each participant to be completed in a classroom setting. Data were analyzed using SPSS 19.0 (Chicago, IL, USA). *P-*values <0.05 were considered statistically significant.

Overall, 158 children with PNE and 168 age- and gender-matched normal healthy children were enrolled in the study. No significant differences were observed between enuretic patients and the control group in terms of demographic data, including gender, age, family history of enuresis, family income, family atmosphere, academic achievement, and family type. Children with PNE had lower total scores and lower scores in one sub-test (behavior test) when compared with their healthy peers (55.23±6.11 vs. 56.60±5.24, 11.79±3.01 vs. 12.41±2.50), and the differences were statistically significant (*P*<0.05, Table 1).

**Table 1:** Comparisons of the children who were and were not diagnosed with PNE in terms of PHCSS subscale scores and total scores

***Variable***	***PNE***	***Normal***	**t**	**P**
Behavior	11.79±3.01	12.41±2.50	2.51	0.014
Intellectual and school status	10.57±3.66	10.89±3.18	1.06	0.102
Physical appearance and attributes	7.53±3.08	7.89±2.85	1.77	0.135
Anxiety	9.35±2.92	9.31±2.54	0.17	0.311
Popularity	8.61±2.17	8.79±1.93	1.01	0.250
Happiness and satisfaction	7.54±1.70	7.60±1.62	0.59	0.283
Total scores	55.23±6.11	56.60±5.24	2.17	0.030

The participants (enuretic patients and normal children) were divided into subgroups according to gender, age, family history of enuresis, family atmosphere, family income, academic performance, and family type. Then total scores of PHCSS of each subgroup were compared. Results revealed that family atmosphere was related with total scores of PHCSS (Table 2).

**Table 2:** Total scores of PHCSS in different subgroups

***Variable***	***Variable***	***N(%)***	***Total scores***	**P**
Gender	Male	167(51.23)	56.38±5.09	0.465
	Female	159(48.77)	56.06±4.78	
Age (yr)	8–11	251(76.99)	56.23±5.00	0.559
	12–15	75(23.01)	56.22±4.76	
Family history of enuresis	NO	197(60.43)	58.39±3.27	0.251
	Yes	129(31.29)	57.11±3.02	
Family atmosphere	Harmonious	240(73.62)	56.83±4.42	0.023
	Moderate	75(23.00)	55.97±4.66	
	Poor	11(3.38)	44.82±3.66	
Family income	Above-average incomes	132(40.49)	56.54±5.59	0.354
	Average incomes	181(55.52)	55.95±4.25	
	Low incomes	13(3.99)	56.92±6.75	
Academic achievement	Excellent	108(33.13)	56.49±5.29	0.260
	Moderate	175(53.68)	55.71±4.75	
	Poor	43(13.19)	57.65±4.55	
Family type	Single-parent	91(27.92)	55.84±5.00	0.323
	Nuclear family	143(43.86)	56.60±4.60	
	Huge family	92(28.22)	56.03±5.37	

Our study demonstrated that children with PNE showed significantly decreased self-concept compared with normal children. The finding was consistent with the results reported by others that adolescents with enuresis scored lower than healthy controls in all six subscales of PHCSS in addition to the total scores (5). Children consider NE as one of the most stressful life events, after parental fighting and divorce or the death of a parent (6). Family atmosphere plays an important role in the mental health of children with NE. This study revealed that adolescents in the group of poor family atmosphere had lower total scores of PHCSS. Poor family atmosphere is not conducive to mental well-being for adolescents with PNE.

Children with PNE exhibit low self-concept compared with their healthy peers, and their emotional status is related with family atmosphere.
